# Induction of immunomodulatory miR-146a and miR-155 in small intestinal epithelium of *Vibrio cholerae* infected patients at acute stage of cholera

**DOI:** 10.1371/journal.pone.0173817

**Published:** 2017-03-20

**Authors:** Aziz Bitar, Rituparna De, Silvia Melgar, Kyaw Min Aung, Arman Rahman, Firdausi Qadri, Sun Nyunt Wai, Tahmina Shirin, Marie-Louise Hammarström

**Affiliations:** 1 Department of Clinical Microbiology, Immunology, Umeå University, Umeå, Sweden; 2 Department of Molecular Biology, The Laboratory for Molecular Infection Medicine Sweden (MIMS), Umeå University, Umeå, Sweden; 3 APC Microbiome Institute, University College Cork, Cork City, Ireland; 4 UCD School of Biomolecular and Biomedical Science, UCD Conway Institute, University College Dublin, Dublin, Ireland; 5 International Centre for Diarrheal Disease Research, Bangladesh, Dhaka, Bangladesh; 6 Department of Virology, Institute of Epidemiology Disease Control & Research (IEDCR), National Influenza Centre (NIC), Dhaka, Bangladesh; Beijing Institute of Microbiology and Epidemiology, CHINA

## Abstract

The potential immunomodulatory role of microRNAs in small intestine of patients with acute watery diarrhea caused by *Vibrio cholerae* O1 or enterotoxigenic *Escherichia coli* (ETEC) infection was investigated. Duodenal biopsies were obtained from study-participants at the acute (day 2) and convalescent (day 21) stages of disease, and from healthy individuals. Levels of miR-146a, miR-155 and miR-375 and target gene (IRAK1, TRAF6, CARD10) and 11 cytokine mRNAs were determined by qRT-PCR. The cellular source of microRNAs in biopsies was analyzed by *in situ* hybridization. The ability of *V*. *cholerae* bacteria and their secreted products to cause changes in microRNA- and mRNA levels in polarized tight monolayers of intestinal epithelial cells was investigated. miR-146a and miR-155 were expressed at significantly elevated levels at acute stage of *V*. *cholerae* infection and declined to normal at convalescent stage (*P*<0.009 versus controls; *P* = 0.03 versus convalescent stage, pairwise). Both microRNAs were mainly expressed in the epithelium. Only marginal down-regulation of target genes IRAK1 and CARD10 was seen and a weak cytokine-profile was identified in the acute infected mucosa. No elevation of microRNA levels was seen in ETEC infection. Challenge of tight monolayers with the wild type V. *cholerae* O1 strain C6706 and clinical isolates from two study-participants, caused significant increase in miR-155 and miR-146a by the strain C6706 (*P*<0.01). One clinical isolate caused reduction in IRAK1 levels (*P*<0.05) and none of the strains induced inflammatory cytokines. In contrast, secreted factors from these strains caused markedly increased levels of IL-8, IL-1β, and CARD10 (*P*<0.001), without inducing microRNA expression. Thus, miR-146a and miR-155 are expressed in the duodenal epithelium at the acute stage of cholera. The inducer is probably the *V*. *cholerae* bacterium. By inducing microRNAs the bacterium can limit the innate immune response of the host, including inflammation evoked by its own secreted factors, thereby decreasing the risk of being eliminated.

## Introduction

*Vibrio cholerae* O1 and Enterotoxigenic *Escherichia coli* (ETEC) are the predominant causes of acute dehydrating diarrhea in developing countries such as Bangladesh where a large proportion of cases requiring hospitalization are reported [1–4]. Both pathogens are non-invasive and colonize the small intestine. Major virulence factors and inducers of diarrhea are the secreted cholera toxin and heat-labile- and heat-stable enterotoxins in the case of *V*. *cholerae* O1 and ETEC, respectively [2, 5]. At the acute stage of disease in *V*. *cholerae* infection an innate immune response is seen at the epithelial lining and its vicinity in the small intestinal mucosa involving accumulation of neutrophil granulocytes, increased production of inflammatory mediators and anti-microbial peptides [6, 7].

MicroRNAs are non-coding, 19–25 nucleotides long, single stranded RNA molecules that regulate inflammation at the post-transcriptional level [8–13]. Although microRNAs on average have the capacity to repress hundreds of target mRNAs, their action as immunomodulators seems to be highly regulated [8, 14–16]. Two microRNAs shown to be highly involved in the regulation of the acute inflammatory response after pathogen recognition through Toll-like receptors (TLRs) are miR-146a and miR-155 [17]. Identified target genes for miR-146a in immune cells are the cytoplasmic adapter molecules Interleukin(IL)-1 receptor-associated kinase 1 (IRAK1) and Tumor necrosis factor(TNF) receptor-associated factor 6 (TRAF6), key molecules in the signaling pathway of Nuclear factor κ-light-chain-enhancer of activated B cells (NFκB) activation downstream of Myeloid differentiation primary response gene 88 (MyD88) after pathogen recognition through TLRs [8, 11, 18–20]. More recently, Caspase recruitment domain family member 10 (CARD10) was identified as a target mRNA for miR-146a upon NFκ B activation of human retinal endothelial cells [21].

Three microRNAs, i.e. miR-146a, miR-155 and miR-375, were shown to be involved in inflammatory conditions of the human intestine. Interestingly, the expression pattern of these microRNAs differed between the chronic inflammatory conditions Crohn's colitis and ulcerative colitis with significantly higher levels of miR-375 at the inflamed compared to non-inflamed areas in Crohn's colitis while miR-146a levels were higher at inflamed areas in ulcerative colitis. Levels of miR-155 were significantly higher in inflamed compared to non-inflamed areas in both diseases [22]. An important role of miR-375 in innate immunity was revealed when it was shown that high levels of miR-375 promote differentiation of intestinal epithelial cells into goblet cells, the producers of the protective mucus layer facing the gut lumen [23]. The potential role of these microRNAs in responses to enteropathogens of the human small intestine is still scarcely investigated, particularly little is known about their role in the innate immune response of the epithelial cells to bacterial attacks.

In the present study we investigated whether *V*. *cholerae* O1 and ETEC infection induce expression of the microRNAs miR-146a, miR-155 and miR-375 in the duodenal mucosa and whether these microRNAs might play a role in resolution of disease by negative regulation of inflammation. Paired biopsies, collected at acute and convalescent stage of disease were analyzed for the expression of miR-146a, miR-155 and miR-375 and their known target genes. The inflammatory status of the mucosa was accessed as expression levels of mRNAs for selected cytokines and chemokines. We also investigated whether *V*. *cholerae* O1, either the whole bacteria or the secreted products, can act as a causative agent for changes in levels of these microRNAs and their known target genes in human intestinal epithelial cells. The results show that miR-146a and miR-155 levels are significantly elevated in *V*. *cholerae* O1 infected patients at acute stage of disease and localized mainly in the epithelium. Furthermore, live *V*. *cholerae* O1 bacteria, but not their secreted products induced all three microRNAs in polarized tight monolayers of intestinal epithelial cells.

## Results

### MicroRNAs miR-146a and miR-155 are expressed in duodenal epithelium of *V*. *cholerae* O1 infected patients

**Fig 1:**
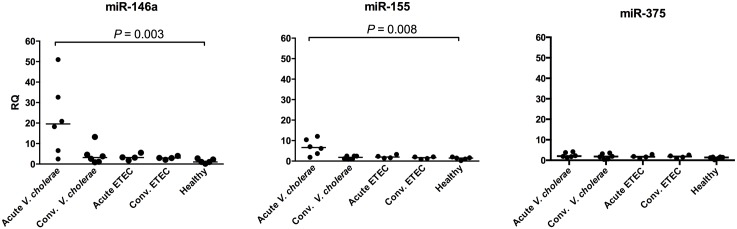
Patients with *Vibrio cholerae* O1 infection at the acute stage show significantly increased levels of microRNAs miR-146a and miR-155. Amounts of miR-146a, miR-155 and miR-375 were determined by real-time qRT-PCR in duodenal biopsies of patients with *V*. *cholerae* O1 infection (n = 6) at the acute (Acute *V*. *cholerae*) and convalescent (Conv. *V*. *cholerae*) stages, patients with ETEC infection (n = 4) at the acute (Acute ETEC) and convalescent (Conv. ETEC) stages and in healthy controls (Healthy; n = 5) and normalized to the amount of RNU48 in the respective sample (Δct). Relative quantity (RQ) was calculated by the 2^(-ΔΔct)^-method with the microRNA/RNU48 Δct-value in the sample over the median microRNA/RNU48 Δct-value of the healthy control group. Each patient group was analyzed for significant changes compared to the healthy controls using Kruskal-Wallis test with Dunn's correction for multiple comparisons. *P*-values for statistically significant differences are given.

To investigate whether the miRNAs miR-146a, miR-155 and miR-375 are induced by *V*. *cholerae* O1 and ETEC infection, paired duodenal biopsies were collected from six *V*. *cholerae* O1 and four ETEC infected patients at acute stage (day-2) and convalescent stage (day-21 post-onset of infection) of disease and compared to single biopsies of five healthy controls. The expression levels of both miR-146a and miR-155 were significantly higher at acute stage of *V*. *cholerae* O1 infection when compared to healthy controls (median relative quantity (RQ) 19.6 and 6.6 for miR-146a and miR-155, respectively; Fig 1) with parallel increase in levels of the two microRNAs in each biopsy (r = 0.943, *P* = 0.017). Notably, no elevated levels were seen for either of these microRNAs in patients with acute ETEC infection (Fig 1). Furthermore, patients in convalescence from *V*. *cholerae* O1 and ETEC infections presented similar levels of miR-146a and miR-155 levels when compared to controls (Fig 1). In contrast to miR-146a and miR-155, the expression level of miR-375 was not significantly elevated either in *V*. *cholerae* O1 or ETEC infected patients (Fig 1).

**Fig 2:**
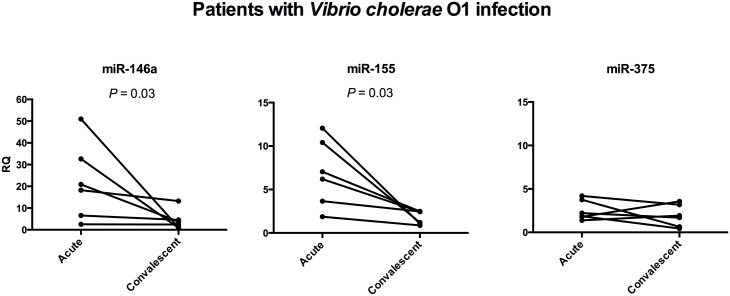
miR-146a and miR-155 levels decrease at the convalescent stage in patients with *V*. *cholerae* O1 infection. Relative quantities (RQ) of miR-146a, miR-155 and miR-375 in duodenal biopsies of patients with *V*. *cholerae* O1 infection (n = 6) in the actue (Acute) and convalescent (Convalescent) state of disease were calculated by the 2^(-ΔΔct)^-method with the microRNA/RNU48 Δct-value in the sample over the median microRNA/RNU48 Δct-value of the healthy control group as reference. Lines connect the RQ-values at acute and convalescent stage of the same patient. Significance of differences between Δct-values at acute and convalescent stage was analyzed using two-sided Wilcoxon's non-parametric test for paired samples. *P*-values for statistically significant differences are given.

Since significantly elevated miR-146a and miR-155 levels were seen in patients with acute *V*. *cholerae* O1 infection, we analyzed the levels in each patient in relation to disease activity, *i*.*e*. in paired samples from acute and convalescent stage. As seen in Fig 2, miR-146a and miR-155 expression levels were significantly higher at the acute stage when compared to the convalescent stage and were decreased to a level similar to that of healthy individuals in each patient after transition to the convalescent stage.

**Fig 3:**
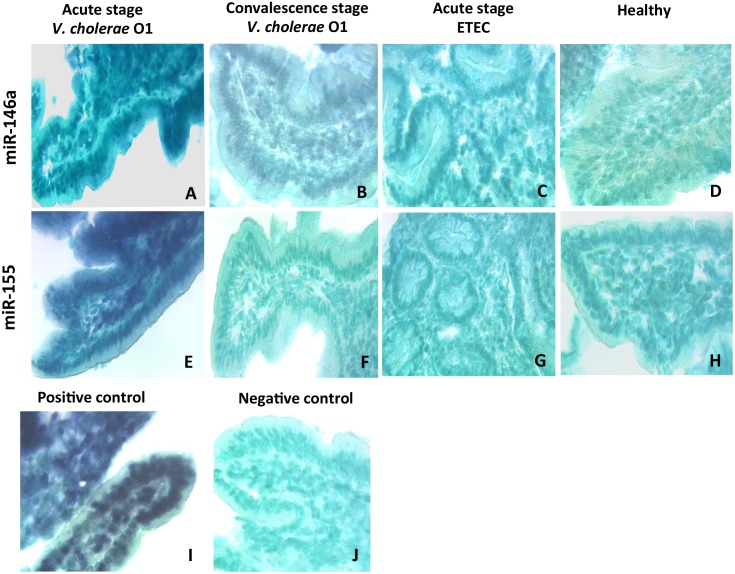
miR-146a and miR-155 are expressed in the epithelium and lamina propria of duodenal mucosa in patients with *V*. *cholerae* O1 infection at the acute stage. *In situ* hybridization was performed on sections of frozen duodenal mucosa using digitonin-labeled locked nucleic acid (LNA) microRNA detection probes. Micrographs are representative of miR-146a (A—D) and miR-155 (E—H) staining of duodenal mucosa from one patient with *V*. *cholerae* O1 infection at acute (A, E) and convalescent (B, F) stage of disease, one patient with ETEC infection at acute stage (C, G) and one healthy control (D, H). I and J show the technical positive and negative control when staining of duodenal mucosa of the patient with *V*. *cholerae* O1 infection at the acute stage with a probe for U6 small nuclear RNA (I) and a scrambled microRNA-probe (J). Original magnification: ×400.

Since miR-146a and miR-155 have mostly been reported in innate immune cells, the cellular source of these microRNAs in the duodenal mucosa at acute stage of *V*. *cholerae* infection was assessed by *in situ* hybridization using probes complementary to these two microRNAs. Hybridization experiments showed that both miR-146a and miR-155 were highly detected in the mucosa of patients with *V*. *cholerae* O1 infection at acute stage with no expression detected in the mucosa from patients with *V*. *cholerae* O1 infection at convalescent stage, at acute or convalescent stage of ETEC infection or in healthy controls (Fig 3A–3H). Notably, at the acute stage of *V*. *cholerae* O1 infection the majority of the cells in the epithelium were positive for miR-146a and miR-155 while only a few scattered positive cells were seen in the lamina propria (Fig 3A and 3E). The intensity of staining for these two microRNAs was comparable to that of the positive control (compare Fig 3A, 3E and 3I). Overall, our data identified increased expression levels of miR-146a and miR-155 in the duodenal epithelium of *V*. *cholerae* O1 infected patients at the acute stage of disease. Based on these findings, the subsequent experiments were focused on this patient group.

### Expression levels of mRNA for the miR-146a target genes IRAK1, TRAF6 and CARD10 do not change with disease activity

**Fig 4:**
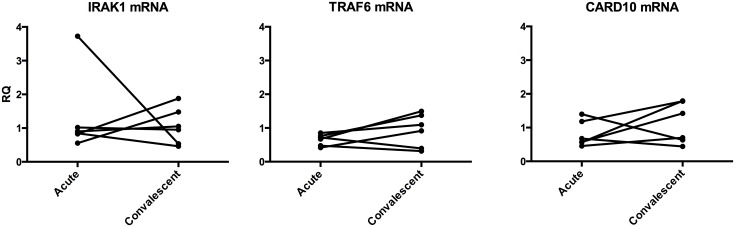
Expression levels of target genes for miR-146a show only marginal differences between the acute and the convalescent stage of disease in patients with *V*. *cholerae* O1 infection. Expression levels of IRAK1, TRAF6 and CARD10 mRNAs were determined in duodenal biopsies collected at acute stage (Acute) and at the convalescent stage (Convalescent) of disease caused by *V*. *cholerae* O1 infection. Expression levels were determined by a real-time qRT-PCR and normalized to the content of 18S rRNA in the sample. Results are shown as relative quantity (RQ) calculated by using the 2^(-ΔΔct)^-method and the median Δct-value at convalescent stage as reference. Each point represents the value of an individual patient at the indicated disease stage. Lines connect the values at acute and convalescent stage of the same patient.

The expression levels of mRNA for three miR-146a targets, namely IRAK1, TRAF6 and CARD10 were analyzed in the duodenal biopsies collected pairwise from patients at acute and convalescent stage of *V*. *cholerae* O1 infection. The results show that all three miR-146a target genes were expressed at significant levels in duodenal samples of patients at acute stage of disease (S1 Table). However, there was no significant difference in their expression levels when these samples were compared to samples from the convalescent stage of disease (Fig 4 and Table 1; *P*>0.05). Still the expression levels of IRAK1, TRAF6 and CARD10 showed the expected increase at convalescent stage in 2, 3 and 4 patients, respectively (Table 2).

**Table 1:** Relative expression levels of mRNAs for miR-146a target genes, chemokines and inflammatory cytokines in duodenal biopsies at acute compared to convalescent stage of disease in patients with *Vibrio cholerae* O1 infection.

mRNA species	Relative quantity at acute stage (RQ)*	*P*-value^#^
	*miR-146a targets*	
IRAK1	0.87 (0.76–1.70)°	>0.99
TRAF6	0.69 (0.46–0.85)	0.44
CARD10	0.62 (0.54–1.24)	0.44
	*Inflammasome cytokines*	
IL-1β	4.17 (2.07–15.38)	**0.06**
IL-18	1.00 (0.57–2.61)	>0.99
	*Chemokines*	
IL-8/CXCL8	5.1 (4.41–6.09)	**0.03**
CXCL9	10.27 (2.60–49.92)	**0.06**
CXCL10	1.30 (0.24–6.12)	0.69
CXCL11	1.19 (0.47–7.21)	0.22
CX3CL1	0.98 (0.55–3.63)	0.84
	*Pro-inflammatory cytokines expressed in epithelial cells and macrophages*	
TNF-α	0.84 (0.50–2.86)	0.69
IL-6	1.44 (0.75–7.58)	0.31
	*Pro-inflammatory cytokines expressed in lymphocytes*	
IL-17A	1.54 (0.92–8.34)	0.44
IFN-γ	1.07 (0.39–4.07)	0.84

* RQ, relative quantity: RQ was calculated as the ratio between the level in the samples at acute stage and the median level of the same mRNA species in the samples at the convalescent stage using the 2^-ΔΔct^-method. Paired biopsies from 6 patients at the acute and the convalescent stage of disease were analyzed.

^#^ *P*-value: The *P*-values were obtained by pairwise comparison of Δct-values at acute and convalescent state of disease using two-sided Wilcoxon test.

° Median and interquartile range (IQR) from the 25^th^ to the 75^th^ percentile.

**Table 2:** Changes in expression levels of mRNA for miR-146a target genes, chemokines IL-8 and CXCL9 and inflammasome cytokine IL-1β from acute to convalescent stage of disease in patients with *V*. *cholerae* O1 infection.

	mRNA levels for miR-146a target genes			
Patient Code	IRAK1	TRAF6	CARD10	Increased relative quantity (RQ) at convalescent stage *
VC08	**↑**	**↑**	**↑**	3/3
VC13	**↑**	**↑**	**↑**	3/3
VC14	**≈**	**↑**	**↑**	2/3
VC16	**≈**	**≈**	**↑**	1/3
VC10	**↓**	**≈**	**↓**	0/3
VC09	**↓**	**↓**	**↓**	0/3
Increased relative quantity (RQ) at convalescent stage°	2/6	3/6	4/6	
	mRNA levels for chemotactic and inflammasome cytokine genes			
Patient Code	IL-8	CXCL9	IL-1β	Decreased mRNA level at convalescent stage^§^
VC08	**↓**	**↓**	**↓**	3/3
VC13	**↓**	**↑**	**↓**	2/3
VC14	**↓**	**↓**	**↓**	3/3
VC16	**≈**	**↓**	**↑**	1/3
VC10	**↓**	**↓**	**↓**	3/3
VC09	**≈**	**↓**	**↓**	2/3
Decreased relative quantity (RQ) at convalescent stage^#^	4/6	5/6	5/6	

**↑**Increased RQ from acute to convalescent stage of disease.

**↓**Decreased RQ from acute to convalescent stage of disease.

**≈** Marginal change in RQ from acute to convalescent stage of disease.

* Number of genes with increased mRNA level at convalescent stage/number of genes analyzed.

° Number of patients with increased mRNA level for the indicated gene/number of patients analyzed.

^§^ Number of genes with decreased mRNA level at convalescent stage/number of genes analyzed.

^#^ Number of patients with decreased mRNA level for the indicated gene/number of patients analyzed.

### The acute stage of *V*. *cholerae* infection induces an epithelial innate immune response

**Fig 5:**
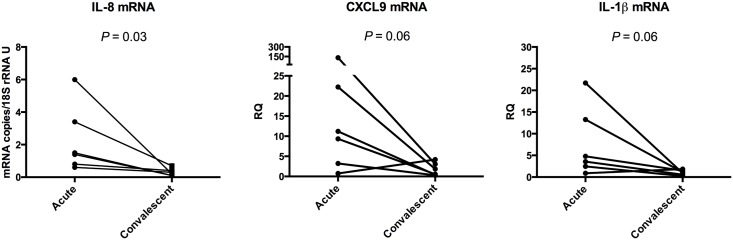
Expression levels of IL-8 mRNA follow disease activity in patients with *V*. *cholerae* O1 infection. Expression levels of IL-8, CXCL9 and IL-1β mRNAs were determined in duodenal biopsies collected at acute stage (Acute) and at the convalescent stage (Convalescent) of disease caused by *V*. *cholerae* O1 infection. The mRNA expression levels were determined by a real-time qRT-PCR and normalized to the content of 18S rRNA in the sample. Results are shown as mRNA copies/18S rRNA U for IL-8 and relative quantity (RQ) calculated by using the 2^(-ΔΔct)^-method and the median Δct-value at convalescent stage as reference for CXCL9 and IL-1β. Each point represents the value of an individual patient at the indicated disease stage. Lines connect the values at acute and convalescent stage of the same patient. Significance of differences between Δct-values at acute and convalescent stage was analyzed using two-sided Wilcoxon's non-parametric test for paired samples and *P*-values are given in the graph.

To investigate the degree of a potential immune response induced by *V*. *cholerae* O1 bacteria in the duodenal mucosa, expression levels of mRNA for the pro-inflammatory cytokines interferon(IFN)-γ, IL-17A, TNF-α, and IL-6; the inflammasome cytokines IL-1β and IL-18; and the immune cell attracting chemokines IL-8, CXCL9, CXCL10, CXCL11 and CX3CL1 were analyzed in the paired duodenal biopsies of the six *V*. *cholerae* O1 infected patients at acute and convalescent stage of disease. Out of the 11 evaluated cytokines only 3, i.e. IL-1β, IL-8 and CXCL9, showed highly increased expression levels in biopsies collected at acute stage of infection when compared to biopsies collected at the convalescent stage (Table 1 and Fig 5). IL-8 had a higher expression level in all six patients at the acute stage although the difference was only marginal in two patients (Table 2 and Fig 5). Levels of CXCL9 mRNA were also higher at the acute stage (Table 1 and Fig 5) followed by a reduced expression level at the convalescent stage in all but one patient (Table 2 and Fig 5). The other 3 chemokines were expressed at low levels and their expression levels were not increased at the acute stage of disease (Table 1 and S1 Table). The average expression level of IL-1β mRNA was higher at the acute stage compared to the convalescent stage and was decreased at convalescent stage in five out of six patients (Tables 1 and 2 and Fig 5). The acute stage of disease did not induce increased mRNA expression levels for the epithelial cell and macrophage derived cytokines TNF-α and IL-6 or for the T lymphocyte derived cytokines IFN-γ and IL-17A (Table 1).

### *V*. *cholerae* O1 bacteria induce increased expression levels of microRNAs in intestinal epithelial cells while secreted products induce an inflammatory response

To address the question whether the bacteria *per se* are the causative agent for the increased microRNA levels seen in the intestinal epithelium during *V*. *cholerae* O1 infection we performed challenge experiments of polarized tight monolayers of the human colon carcinoma cell line T84. *V*. *cholerae* O1 isolates from two of the patients included in this study, patient-codes VC08 and VC09, as well as the wild-type of *V*. *cholerae* O1 strain C6706 were used for the challenge experiments. Bacteria and culture supernatants were applied at the apical side in order to mimic the conditions during intestinal infection in the patients.

**Fig 6:**
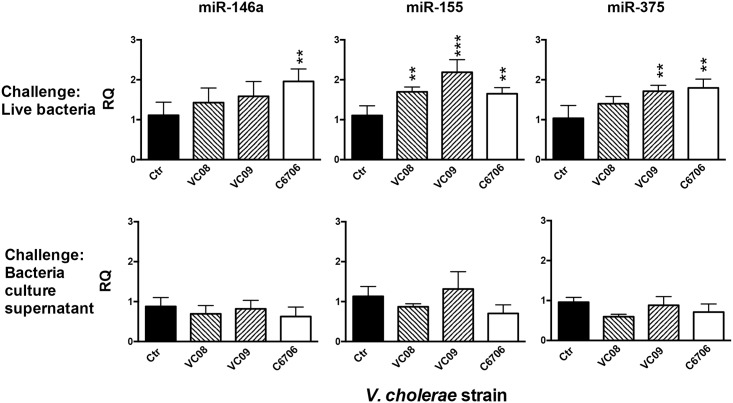
*V*. *cholerae* O1 bacteria, but not their secreted products, induce increased expression of microRNAs in T84 polarized tight monolayer cells. Tight monolayers of polarized T84 cells were challenged at the apical side with 10^5^ bacteria of the *V*. *cholerae* O1 wild type strain C6706 and two *V*. *cholerae* O1 clinical isolates, one from patient VC08 (VC08) and one from patient VC09 (VC09), that had been grown in LB broth overnight (upper row) or with culture supernatants from overnight cultures of the same bacterial strains in LB (lower row). Levels of miR-146a, miR-155 and miR-375 were determined by real-time qRT-PCR and expressed as relative quantity (RQ) compared to the median Δct of tight monolayers incubated in parallel to challenged monolayers with tissue culture medium without added bacteria (Ctr, upper row) or fresh LB (Ctr, lower row). Bars indicate mean RQ + 1 SD of 4 tight monolayers for each challenge in the upper row and 3 in the lower row. Statistically significant differences from the control monolayers as determined by one-way ANOVA with Dunett's compensation for multiple comparisons, are shown. ** *P*-value <0.01, *** *P*-value <0.001.

**Fig 7:**
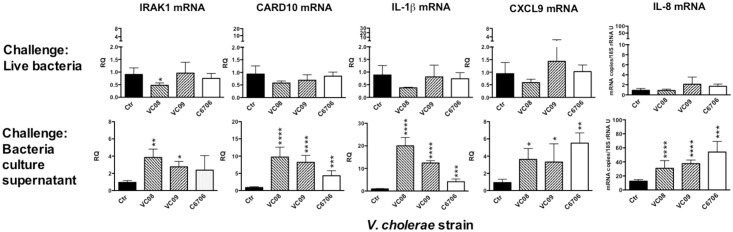
Secreted products of *V*. *cholerae* O1 bacteria induce increased expression of genes involved in inflammation in T84 polarized tight monolayer cells while live bacteria do not. The same RNA samples that were analyzed for miRNA expression levels (results shown in Fig 6) were also analyzed for expression levels of mRNAs for the microRNA target genes IRAK1 and CARD10, the inflammasome cytokine IL-1β and the chemokines CXCL9 and IL-8. The mRNA expression levels were determined by a real-time qRT-PCR and normalized to the content of 18S rRNA in the sample. Results are shown as relative quantity (RQ) calculated by using the 2^(-ΔΔct)^-method and using the median Δct-value of the sham-treated controls as reference for IRAK1, CARD10, IL-1β and CXCL9 and as mRNA copies/18S rRNA U for IL-8. Bars indicate mean + 1 SD (n = 4 in upper row and n = 3 in lower row). Statistically significant differences from the sham-treated control monolayers as determined by one-way ANOVA with Dunett's compensation for multiple comparisons, are shown. * *P*-value <0.05; ** *P*-value <0.01; *** *P*-value <0.001; **** *P*-value <0.0001.

Challenge with live bacteria, of both the clinical isolates and the C6706 wild-type strain, induced expression microRNAs in the polarized epithelial cells. Similarly, bacteria that had been grown in LB broth, *i*.*e*. the growth conditions under which both *V*. *cholera* protease V (PrtV) and cytolysin (VCC) are produced [24, 25], induced all three microRNAs tested. The wild type C6706 strain induced statistically significant increased expression levels of all three microRNAs, while the clinical strains VC08 and VC09 induced significant increase of miR-155 levels (Fig 6, upper row). The latter two strains also induced increased levels of miR-146a although the values did not reach statistical significance (Fig 6, upper row; *P*>0.05). Bacteria that had been grown in AKI broth, *i*.*e*. growth conditions under which cholera toxin (CT) is produced (S1 Fig) [26] induced only miR-375 (S2 Fig, upper row). In contrast to the results obtained with live bacteria, culture supernatants collected from the three *V*. *cholerae* O1 strains grown in either LB or AKI broth did not cause significant changes in microRNA levels in the tight monolayer cells (Fig 6, lower row and S2 Fig, lower row). Next, we analyzed the mRNA expression levels of the microRNA target genes IRAK1 and CARD10 after challenge with bacteria grown in LB. TRAF6 was not included since this mRNA species is barely detected in T84 monolayer cells (S2 File) and hence it would not be possible to detect a further down-regulation. Only the live bacteria of the VC08 strain caused statistically significant reduction of IRAK1 mRNA levels (Fig 7, upper row). Whole bacteria of the same strain did induce significant increase of only one microRNA species, namely miR-155 (Fig 6, upper row). In contrast, and somewhat unexpected, LB culture supernatants from the VC08 strain caused significant increase in IRAK1 mRNA levels (Fig 7, lower row). In line with this, LB culture supernatants from the three bacterial strains all caused significant increase in levels of CARD10 mRNA (Fig 7, lower row). Another unexpected finding was that challenge with live bacteria did not cause any inflammatory response monitored as increased expression levels of IL-1β, CXCL9 and IL-8 (Fig 7). In contrast, culture supernatants from all three strains cause significant increases in expression levels of mRNA for all three cytokines (Fig 7, lower row).

## Discussion

In the present study we identified the increased expression of miR-146a and miR-155 in a major population of epithelial cells and in scattered cells, presumably immune cells, in the lamina propria of duodenal mucosa in patients at the acute stage of *V*. *cholerae* O1 infection, while these microRNAs were not induced by ETEC infection. Furthermore, we show that *V*. *cholerae* O1 bacteria, including clinical isolates from patients that we demonstrated increased microRNA expression in the duodenal epithelium, induced an increased expression of miR-146a, miR-155 and miR-375 in T84 polarized tight monolayer cells, suggesting that *V*. *cholerae* O1 bacteria can indeed be the cause of the increased levels of miR-146a and miR-155 in the epithelium of the infected patients. To the best of our knowledge this is the first time that increased levels of miR-146a and miR-155 have been demonstrated in the small intestinal mucosa of *V*. *cholerae* O1 infected patients and the first time that *V*. *cholerae* O1 bacteria have been shown to cause increased levels of these microRNAs in intestinal epithelial cells. Whether the difference between *V*. *cholerae* O1 and ETEC infected patients is due to different properties of the two bacterial species or merely is a consequence of a small sample size in the ETEC group (n = 4) remains to be elucidated.

*V*. *cholerae* O1 bacteria caused increased levels of miR-375 in T84 tight monolayer cells, yet the miR-375 levels were not increased in the duodenal mucosa of the *V*. *cholerae* O1 infected patients. This discrepancy might, at least in part, reflect organ-specific differences between epithelial cells in small and large intestine since T84 cells originate from colonic epithelium. There are however, additional factors that could contribute to differences in microRNA expression in the duodenal mucosa of patients and the T84 tight monolayers, which can be difficult to accurately mimic in an *in vitro* model. Firstly, the time during which the epithelium is exposed to bacteria and their factors, days *in vivo* in the patients compared to a merely 5 hours co-culture in the *in vitro* model. Secondly, the duodenal mucosa is rich in immune cells and inflammatory mediators such as cytokines and chemokines secreted by them might influence the function(s) of the epithelial cells and their readiness to modulate microRNA expression levels. Immune cells are not present in the *in vitro* model. Thirdly, the bacteria might differ in their capacity to induce microRNA expression due to properties regulated by their environment. Thus, we found that the same *V*. *cholerae* O1 bacterial strains induced different microRNAs depending on which broth they were grown in, *i*.*e*. mainly miR-155 was observed after growth in LB broth, in which the bacteria secrete both VCC and PrtV and mainly miR-375 induction was observed after growth in AKI broth, which promotes CT secretion. How the environment at the epithelial lining of the patients with acute cholera is influencing the capacity of *V*. *cholerae* O1 bacteria to induce different microRNAs is currently unknown.

While miR-146a and miR-155 tend to shutdown innate immunity, miR-375 promotes the differentiation of intestinal epithelial cells into goblet cells [23], which is also an important arm of the innate immune defence. By increasing the proportion of goblet cells with consequent increased production of mucus the mucosa might be protected by preventing bacteria from reaching the epithelial cell surface. In the colon the proportion of goblet cells in the epithelium is higher, the mucosal layer is thicker and the bacterial load is much larger than in the small intestine. This might be an as to explanation for why T84 cells with colonic origin are prone to increase levels of miR-375.

The expression of IRAK1 and TRAF6 was only down-regulated in 2 and 3 out of 6 cholera patients at the acute stage of disease although miR-146a and miR-155 were expressed at high levels. This apparently contradictory result was also seen in rheumatoid arthritis. Elevated levels of miR-146a was found in synovial fibroblast and blood mononuclear cells of rheumatoid arthritis patients while the levels of IRAK1 and TRAF6 mRNAs were similar to those of controls [18, 27]. The counteracting effects, anti-inflammatory from the bacteria themselves and pro-inflammatory from their secreted products, may be the explanation for the unchanged levels of IRAK1 and TRAF6 mRNAs in patients with acute *V*. *cholerae* infection.

The increased expression levels of IL-8, IL-1β and CXCL9 mRNAs in the duodenal mucosa at the acute stage of disease suggest an on-going local inflammation in the small intestine. However, the magnitude of this inflammatory response was rather weak when estimated as cytokine mRNA levels at acute compared to convalescent stage. The elevated levels of the neutrophil chemoattractant IL-8 at acute stage is consistent with previous findings of larger amounts of IL-8 secreted by lamina propria cells and accumulation of neutrophils in the duodenal mucosa at the acute phase [6, 28]. Elevated levels of IL-8 are also in agreement with increased production of IL-8 by tight monolayers of epithelial cells after challenge with culture supernatant of *V*. *cholerae* O1 bacteria [24, 25]. However, challenge of polarized tight monolayers with whole *V*. *cholerae* O1 bacteria failed to induce increased levels of IL-8. Instead challenge with bacteria applied at the side from which they would have attacked *in vivo*, induced significant increases in both miR-146a and miR-155 levels. Taken together these data suggest that the epithelium receives contradictory signals from the bacteria during infection meaning pro-inflammatory from secreted factors and inflammation-limiting from the bacterial cells. The variation in magnitude of inflammatory response between individuals might reflect the outcome from differences in amount and composition of factors secreted by the bacteria and differences in the mucus barrier function of the patient yielding varying degree of access of the bacteria to the epithelial surface.

Levels of the lymphocyte and monocyte chemoattractant CXCL9 and the inflammasome-activated cytokine IL-1β were higher at acute stage compared to the convalescent stage (*P* = 0.06), but the response was relatively weak. This latter finding is in line with the proposed down-regulatory role of miR-146a and miR-155 on inflammation and cytokine production [8, 18, 20, 29] and suggests that these microRNAs indeed limit the inflammatory response to *V*. *cholerae* O1 bacteria during infection. In agreement with this, *H*. *pylori* infection up-regulates the levels of miR-146a and miR-155 and down-regulates of the pro-inflammatory cytokines IL-8 and growth-related oncogene (GRO)-α in the human gastric mucosa [29, 30].

In the present study we did not see disease activity related changes in expression of TNF-α and IL-6, for which the main cellular sources are monocytes/macrophages and epithelial cells. These results are in apparent contradiction to previous analyses of lamina propria cells at the protein level [6, 28].

Two T lymphocyte derived cytokines were also analyzed in this study, IFN-γ and IL-17A. They were chosen because T-helper cells of individuals who had gained immunity through natural infection produced these cytokines upon *in vitro* challenge with *V*. *cholerae* antigens [28]. Furthermore, they are the dominating cytokines produced by T lymphocytes in the inflamed small intestinal mucosa of patients with active celiac disease [31, 32]. The levels of these T cell cytokines were low and no changes related to disease activity were observed. The results on IL-17A are concordant with a previous study at the protein level [28]. Overall, these results suggest that the immune response at acute stage of cholera is mainly innate and to a large extent emanates from epithelial cells.

**Fig 8:**
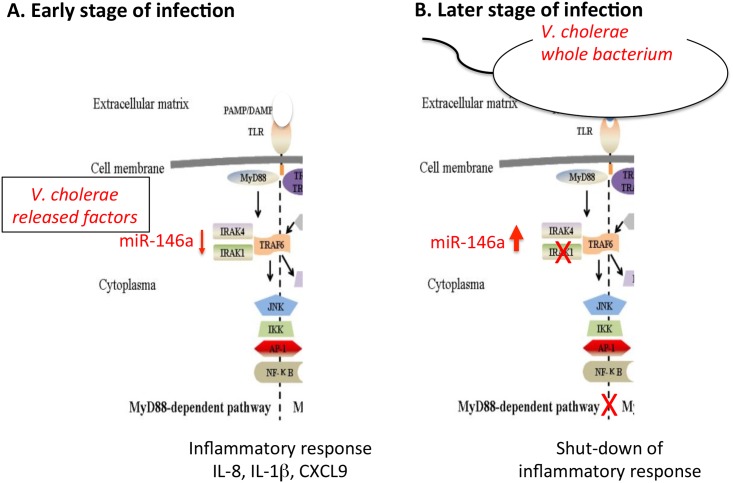
Hypothetical scenario of what is happening at the epithelial lining of the small intestine at the acute phase of *V*. *cholerae* infection. *V*. *cholerae* is a Gram(-), flagellated bacterium that secretes several soluble factors, e.g. cholera toxin and *V*. *cholerae* cytolysin, and release outer-membrane vesicles (OMVs). The released components are all potential inducers of inflammatory responses. We hypothesize that the bacteria secrete these factors already in the gut lumen. Their soluble nature makes it possible for them to pass through the mucous layer, reach the epithelial surface and enter into the cytoplasm. The epithelial cells respond to this invasive attack by activating the IL-1β inflammasome and secretion of chemokines recruiting immune cells to the site, notably IL-8 a strong attractant for neutrophil granulocytes (A). The *Vibrio cholerae* bacterium is motile and can produce mucinases, hence it would be feasible for the bacteria to penetrate the mucous layer and reach the surface of the epithelial cells, although this process would be slower than for the released factors. Thus, the bacterium reaches the epithelium with an ongoing innate immune response. By binding to receptors at the cell surface the bacterium induces increased expression of microRNAs miR-146a and miR-155, thereby decreasing levels of IRAK1 with a consequent decreased inflammatory activity and decreased risk for the bacterium to be eliminated and increasing its chances to thrive at the epithelial lining and even get access to the bodily tissues (B). Figure adapted from: J Zhong, J-F Xu, P Yang, Y Liang, C-Y Wang (2011). Innate Immunity in the Recognition of β-Cell Antigens in Type 1 Diabetes, Type 1 Diabetes—Pathogenesis, Genetics and Immunotherapy, Prof. David Wagner (Ed.), ISBN: 978-953-307-362-0, InTech.

Taken together the results suggest that *V*. *cholerae* O1 secretes factor(s) that induce an inflammatory response in intestinal epithelial cells and that the bacteria themselves have the capacity to counteract this response by inducing microRNAs that limit the inflammatory response thereby reducing the risk for the bacteria to be eliminated by the host. A possible scenario for the events occurring at acute stage of *V*. *cholera* infection is outlined in Fig 8. At the earliest stage of infection, bacteria distant from the epithelial surface release components that, when reaching the epithelium enter into the cytoplasm of the epithelial cells and provoke an inflammatory response via the NFκB-pathway. The response includes increased expression of the mediators IRAK1 and CARD10, and induction of pro-inflammatory chemokines and cytokines such as IL-8, CXCL9 and IL-1β (Fig 8A). Previous studies have shown that both soluble factors and outer membrane vesicles released by *V*. *cholerae* can cause activation of NFκB and production of pro-inflammatory cytokines [25, 33, 34]. At a later stage (Fig 8B), when the bacterium itself has passed the mucous layer and reached the epithelium it might bind to receptors, e.g. TLRs, at the cell surface causing intracellular signaling with consequent increased levels of miR-146a causing decreased levels of target genes such as IRAK1 and subsequent shut-down of inflammation including the cessation of chemokine IL-8 and CXCL9 production leading to reduced recruitment of immune cells to the infected site. This study suggest that *V*. *cholerae* O1 has captured part of the host mechanism for ending an innate immune reaction of the epithelium by acquiring the capacity to induce increased levels of the inflammatory regulators miR-146a and miR-155. It is tempting to speculate that enteropathogens that have this capacity can cause more severe disease than those who do not. The nature of these factor(s) remains to be elucidated.

## Materials and methods

### Study groups and sample collection

Ten adult male patients, aged 22–46 years, with culture confirmed *V*. *cholerae* O1 (n = 6) and ETEC (n = 4) infection were included in the study. Paired duodenal biopsies from the 2^nd^ part of the duodenum were collected from each patient at the acute stage (day 2 post-onset of disease) and at the convalescent stage (day 21 post-onset of disease) of infection by using a standard endoscope (Olympus, Tokyo, Japan). Duodenal biopsies were also collected from healthy adults, defined as individuals who did not have a history of diarrheal illness in the previous 3 months (n = 5). Biopsies were directly stored in Trizol (Invitrogen Life Technologies, Carlsbad, CA, USA) for analysis of mRNAs and microRNAs by real-time quantitative reverse transcriptase-polymerase chain reaction (qRT-PCR) and also embedded in Optimal cutting temperature compound (OCT-Tissue-Tek; Miles, Elkhart, IN, USA) for *in situ* hybridization. All patients enrolled in this study were admitted to International Centre for Diarrheal Diseases Research, Bangladesh (iccdr,b) with moderate to severe degree of dehydration. The ethical review committee of iccdr,b approved the study. All participants gave their written informed consent to participate in the study.

### Bacterial strains

*V*. *cholerae* O1, El Tor, Inaba strain C6706, and *V*. *cholerae* O1, Ogawa, strains isolated at diagnosis [7] from the patients with codes VC08 and VC09. Strains were frozen and stored at -80°C in Luria-Bertani (LB) broth containing 15% glycerol. For polarized tight monolayer challenge experiments, the bacteria were grown overnight at 37°C with shaking in 10 mL of LB or AKI broth. Bacterial cells were harvested by centrifugation at 14,000 rpm for 3 min at 4°C. The pellets were re-suspended in 1 mL tissue culture medium and the supernatants were sterile filtered through a 0.22-μm PVDF membrane filter (Millipore, Merck Chemicals and Life Science, Solna, Sweden).

### Culture of T84 cells and establishment of polarized tight monolayers

The established colon carcinoma cell line T84 (American Type Culture Collection, Rockville, MD, USA) was cultured at 37°C in a humidified atmosphere with 5% CO_2_. Tissue culture medium used was a 1:1 mixture of Dulbecco's modified Eagle's medium and Ham’s F12 medium supplemented with 15 mM HEPES buffer, 8% fetal calf serum, 2 mM L-glutamine, 100 units (U)/mL penicillin and 85μg/mL streptomycin. All tissue culture media components were from Invitrogen (Paisley, UK). Confluent cultures were trypsinized by incubation for 5 min at 37°C with 0.25% trypsin and 0.5% EDTA in phosphate buffered saline (PBS; pH 7.2). After trypsinization cells were allowed to recover for 60 min at 37°C in complete medium.

Tight monolayers were established by seeding 0.5×10^6^ T84 cells in 0.5 mL complete culture medium per well in transwell inserts with semi-permeable polycarbonate membrane supports with 12 mm diameter and 0.4 μm pore size (Costar 3401; Corning Incorporated, Corning, NY, USA). Complete culture medium (1.5 mL) was also added to the outside of the inserts. From the second day after seeding, medium was changed every day until a confluent monolayer with a transepithelial electrical resistance of ≥ 700 Ohm/cm^2^ was obtained [35]. Transepithelial electrical resistance was measured by using the Millicell Electrical Resistance System (Millipore) with chopstick electrodes.

Challenges with whole bacteria and bacterial products were done from the apical side of the polarized tight monolayers. Tissue culture medium in the upper chamber was replaced by either 0.5 mL of culture supernatant obtained from overnight culture of *V*. *cholerae* O1 strain C6706 and two clinical isolates *V*. *cholerae* O1 from patients participating in this study, VC08 and VC09, in LB or AKI broth or 0.5 mL tissue culture medium containing 10^5^ bacteria. Bacteria had grown overnight in LB or AKI and were thereafter retrieved by centrifugation. Tight monolayers to which fresh LB, fresh AKI, or tissue culture medium had been added to the apical side served as controls. Challenged tight monolayers and control tight monolayers were set up in triplicates or quadruplicates and were incubated in parallel for 5 hours at 37°C in humidified atmosphere with 5% CO_2_. Finally the membranes with tight monolayers were cut out, washed 2 times with RNase-free PBS, frozen in 700 μL RLT buffer (pH 7.0) of the RNeasy Mini Kit (Qiagen Valencia, CA, USA) with 1% 2-mercaptoethanol added and kept at -80°C until RNA extraction.

### RNA extraction

Total RNA was extracted from Trizol preserved biopsy samples according to manufacturer’s instructions (Invitrogen) and purified on RNeasy columns (Qiagen) [36]. Possible contaminating genomic DNA was removed by DNase I treatment [36].

Total RNA was extracted from T84 tight monolayers frozen in RTL buffer with 1% 2-mercaptoethanol by using the RNeasy Mini Kit (Qiagen) and dissolved in RNase-free water. RNase inhibitor, recombinant RNasin (1000 U/mL; Promega, Madison, WI, USA), was added to each sample and samples were stored at -80°C [37].

### Determination of expression levels of microRNAs by using real-time qRT-PCR

Expression levels of microRNAs miR-146a, miR-155 and miR-375, and small nuclear RNA RNU48 were determined by real-time qRT-PCR using the TaqMan MicroRNA Reverse Transcription (RT) Kit and TaqMan MicroRNA (TM) Assay primers for human miR-146a (hsa miR-146a: 000468), miR-155 (hsa-miR-155: 002623), miR-375 (hsa-miR-375: 000564), and RNU48 (RNU48: 001006) (Life Technologies, Foster City, CA, USA). RNA concentration was determined by measuring optical density (OD) at 260 nm and purity checked as the OD_260_/OD_280_ ratio where ratios above 1.95 were accepted (Nanodrop spectrophotometer). Ten ng RNA was used for each sample in each real-time qRT-PCR reaction. The samples were analyzed in duplicates using the ABI prism 7900 HT sequence detection system (Applied Biosystems, Foster City, CA). Expression levels were estimated by calculating the relative quantity (RQ) by using the 2^(-ΔΔct)^-method [38]. The levels of microRNAs were normalized to RNU48 by calculating the Δct between the ct-value of the microRNA species and the ct-value of the RNU species in the same sample. ΔΔct was thereafter calculated as the microRNA/RNU Δct-value in the sample minus the median microRNA/RNU Δct-value of either the healthy control group or the patient group with *V*. *cholerae* infection at convalescent stage and in the case of tight monolayers the Δct-value in the sample minus the median microRNA/RNU Δct-value of control T84 tight monolayers sham-treated in parallel to tight monolayers challenged with *V*. *cholerae* O1 bacteria or their secreted products.

### Determination of expression levels of target-gene, cytokine and chemokine mRNAs by using real-time qRT-PCR

Quantification of IL-8/CXCL8, CX3CL1, TNF-α, IL-6, IL-17A, and IFN-γ mRNAs was performed using real-time qRT-PCR assays developed in the laboratory at Immunology, Umeå University. The assays are based on the EZ-technology, with primers placed in different exons, a reporter dye marked probe placed over the exon boundary in the amplicon and use of an RNA copy standard. For details of the IL-8/CXCL8 and IL-6 assays see Ou et al. [35], the TNF-α and IFN-γ assays Forsberg et al. [31], the IL-17A assay West et al. [39] and the CX3CL1 assay Sjöberg et al. [40]. Results are given as mRNA copies/μl. Quantification of IRAK1, TRAF6, CARD10, IL-1β, IL-18, CXCL9, CXCL10, and CXCL11, mRNAs was performed using the Taqman gene Expression Assays Hs01018347_m1, Hs00377558_m1, Hs00367225_m1, Hs00174097_m1, Hs_01038788_m1, Hs00970538_m1, Hs00171042_m1, and Hs00171138_m1, respectively (Applied Biosystems). The concentration of 18S rRNA was determined in each sample using real-time qRT-PCR (Applied Biosystems) and expressed as arbitrary units from a standard curve of serial dilutions of a preparation of total RNA from human peripheral blood mononuclear cells. One U was defined as the amount of 18S rRNA in 1 pg of RNA, and corresponds to approximately 1 epithelial cell [41]. All samples included in the study contained >16 U 18S rRNA per reaction mixture.

Samples were analyzed in triplicates using the ABI prism 7900 HT sequence detection system (Applied Biosystems). mRNA concentrations were normalized to the18S rRNA concentration in the sample by calculating mRNA copies/18S rRNA unit for mRNAs analyzed by assays with an RNA copy standard and by calculating the Δct between the ct-value for the mRNA species and the ct-value for 18S rRNA (ct_sample_−ct_18S rRNA_) for mRNAs analyzed by commercial Taqman gene Expression Assays. Since there is no standard for the Taqman gene Expression Assays result from these analyses are given as relative quantity (RQ) calculated by the 2^(-ΔΔct)^-method where ΔΔct is Δct for the sample minus the median of the Δct-values of the patient group with *V*. *cholerae* infection at convalescent stage or control T84 tight monolayers sham-treated in parallel to monolayers challenged with *V*. *cholerae* bacteria or their secreted factors.

### *In situ* microRNA hybridization

*In situ* hybridization was performed on 12 μm thick cryostat sections on SuperFrost Plus glass slides (Thermo Fisher Scientific Gerhard Menzel, Braunschweig, Germany) obtained from duodenal biopsies and fixed in 4% paraformaldehyde. Digoxigenin (Dig)-labeled miRCURY locked nucleic acid (LNA) microRNA detection probes from Exiqon (Vedbaek, Denmark) were used for the detection of microRNAs in the duodenal mucosa. The probes used were the miR-146a complementary probe hsa-miR-146a (sequence: /5DigN/AACCCATGGAATTCAGTTCTCA/2Dig_N/), the miR-155 complementary probe hsa-miR-155 (sequence: /5DigN/ACCCCTATCACGATTAGCATTAA/3Dig_N/), the RNU6 complementary probe hsammurno (positive control, sequence: /5DigN/ACCCCTATCACGATTAGCATTAA/3Dig_N/), and Scramble-miR (negative control, sequence: /5DigN/GTGTAACACGTCTATACGCCCA/3Dig_N/). The probes were diluted in hybridization solution [500 mL Formamide, 200 mL 20xSSC, 50 mL of 20×Denhardt’s solution, 50 mL of denatured herring sperm DNA (10mg/ mL), 25 mL Bakers yeast RNA (10 mg/ mL) and 175 mL of 50% Dextran sulfate]. Tissue sections were incubated with diluted probes overnight at 60°C in a humid chamber. After hybridization bound probes were revealed by incubation with alkaline phosphatase conjugated Fab-fragments of anti-Dig antibodies (Roche, Mannheim, Germany) and subsequent color reaction was carried out by using the NBT/BCIP reagent (Roche). Tissue sections were counterstained with methylgreen. Microscopic evaluation was performed with a standard light microscope equipped with 3CCD camera (Hamamatsu photonics, Hamamatsu City, Japan). Leica QWin software (Leica Imaging System, Cambridge, UK) was used for image acquisition.

### Statistical analyses

Descriptive data are expressed as median values with the 25^th^ and 75^th^ percentiles, interquartile range (IQR) for analyses performed on clinical material and mean ± 1 standard deviation (SD) for tight monolayer experiments. Statistical analyses on changes in microRNA and mRNA expression levels were performed using the normalized microRNA and mRNA amounts obtained by calculating the Δct-values between ct-values of microRNA species under investigation and the ct-value for RNA48 in the same sample and between the ct-value of the mRNA species under investigation and the ct-value of 18S rRNA in the same sample, respectively. Statistical analysis was performed comparing expression levels as mRNA copies/18S rRNA U for mRNAs for which a qRT-PCR assay with mRNA copy standard was available. Statistical analysis of differences in microRNA and mRNA levels in samples from acute and convalescent stage of *V*. *cholerae* infection was performed using the paired, non-parametric Wilcoxon t-test. Comparisons between microRNA levels between more than two data sets from analysis of patient material was performed using Kruskal-Wallis' nonparametric one-way analysis of variance (ANOVA) with Dunn’s multiple comparisons post-test. Correlation analysis comparing miR-146a and miR-155 levels in biopsies were was performed using Spearman correlation analysis test. Statistical analysis of differences in microRNA and mRNA levels in polarized tight monolayers challenged with *V*. *cholerae* bacterial strains or their secreted products, was performed using ordinary one-way ANOVA with Dunett's multiple comparison post-test. Statistical analyses were performed using the Prism 5 computer program (GraphPad Software, San Diego, CA, USA). Two-sided analysis was used throughout. A *P*-value <0.05 was regarded as statistically significant.

## Supporting information

S1 Fig *Vibrio cholerae* O1 bacteria clinical isolates VC08 and VC09 secrete both Cholera Toxin (CT) and *V*. *Cholerae* Cytolysin (VCC) when grown in AKI broth. Western-blot analyses to detect *V*. *cholerae* cholera toxin (CT) (a, c) or *V*. *cholerae* cytolysin (VCC) (b, d) in overnight culture supernatants of the wild-type *V*. *cholerae* O1 strain C6706 (C6706/WT), *V*. *cholerae* O1 strain C6706 CT deletion mutant (C6706/Δ*CT*), *V*. *cholerae* O1 strain C6706 VCC deletion mutant (C6706/*ΔVCC*) and two *V*. *cholerae* O1 clinical isolates, one from patient VC08 (VC08) and one from patient VC09 (VC09), that had been grown in AKI broth overnight. (a) and (b) show Western-blots using anti-CT and anti-VCC antibodies, respectively. (c) and (d) show semiquantitative analyses of CT (c) and VCC (d) protein levels obtained from chemiluminescence analysis of the same samples. Lane 1 and 5 (VC08); Lane 2 and 6 (VC09); Lane 3 and 8 (C6706/Δ*CT*); Lane 4 and 9 (C6706/WT); and Lane 7 (C6706/Δ*VCC*). The arrow shows the immunoreaction bands of CT or VCC protein. The plotted data in (c) and (d) show relative protein levels of CT (c) and VCC (d) compared to the levels of the respective protein in culture supernatant of the wild-type C6706 strain. Y-axis in (c) and (d) = relative level of protein, with the level of the wild-type of the strain C6706 set to 1.0.
(TIF)

S2 Fig *Vibrio cholerae* O1 bacteria, but not their secreted products, induce increased expression of miR-375 microRNA in T84 polarized tight monolayer cells. Tight monolayers of polarized T84 cells were challenged at the apical side with 10^5^ bacteria of the *V*. *cholerae* O1 strain C6706 (C6706) and two *V*. *cholerae* O1 clinical isolates, one from patient VC08 (VC08) and one from patient VC09 (VC09), that had been grown in AKI broth overnight (upper row) or with culture supernatants from overnight cultures of the same bacterial strains in AKI (lower row). Levels of miR-146a, miR-155 and miR-375 were determined by real-time qRT-PCR and expressed as relative quantity (RQ) compared to the median Δct of tight monolayers incubated in parallel to challenged monolayers with tissue culture medium without added bacteria (Ctr, upper row) or fresh AKI (Ctr, lower row). Bars indicate mean RQ + 1 SD of 4 tight monolayers for each challenge in the upper row and 3 in the lower row. Statistically significant differences from the control monolayers as determined by one-way ANOVA with Dunett's compensation for multiple comparisons, are shown. * *P*-value <0.05, ** *P*-value <0.01, *** *P*-value <0.001.
(TIFF)

S1 Table Expression levels of mRNAs for miR-146a target genes, chemokines and inflammatory cytokines in duodenal biopsies at acute compared to convalescent stage of disease in patients with *Vibrio cholerae* O1 infection. (DOCX)

S1 File Supplementary materials and methods. (DOCX)

S2 File Supplementary data. (XLSX)
