# Supplementary figures and images for: Induction of immunomodulatory miR-146a and miR-155 in small intestinal epithelium of Vibrio cholerae infected patients at acute stage of cholera
Source: PLoS One. 2017 Mar 20;12(3):e0173817. doi: 10.1371/journal.pone.0173817 (PMC5358779; doi:10.1371/journal.pone.0173817)

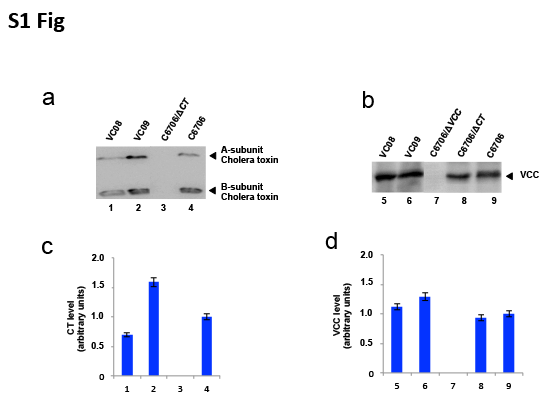

Supplement: S1 Fig — Western-blot analyses to detect V. cholerae cholera toxin (CT) (a, c) or V. cholerae cytolysin (VCC) (b, d) in overnight culture supernatants of the wild-type V. cholerae O1 strain C6706 (C6706/WT), V. cholerae O1 strain C6706 CT deletion mutant (C6706/ΔCT), V. cholerae O1 strain C6706 VCC deletion mutant (C6706/ΔVCC) and two V. cholerae O1 clinical isolates, one from patient VC08 (VC08) and one from patient VC09 (VC09), that had been grown in AKI broth overnight. (a) and (b) show Western-blots using anti-CT and anti-VCC antibodies, respectively. (c) and (d) show semiquantitative analyses of CT (c) and VCC (d) protein levels obtained from chemiluminescence analysis of the same samples. Lane 1 and 5 (VC08); Lane 2 and 6 (VC09); Lane 3 and 8 (C6706/ΔCT); Lane 4 and 9 (C6706/WT); and Lane 7 (C6706/ΔVCC). The arrow shows the immunoreaction bands of CT or VCC protein. The plotted data in (c) and (d) show relative protein levels of CT (c) and VCC (d) compared to the levels of the respective protein in culture supernatant of the wild-type C6706 strain. Y-axis in (c) and (d) = relative level of protein, with the level of the wild-type of the strain C6706 set to 1.0. (TIF) [file pone.0173817.s001.tif]

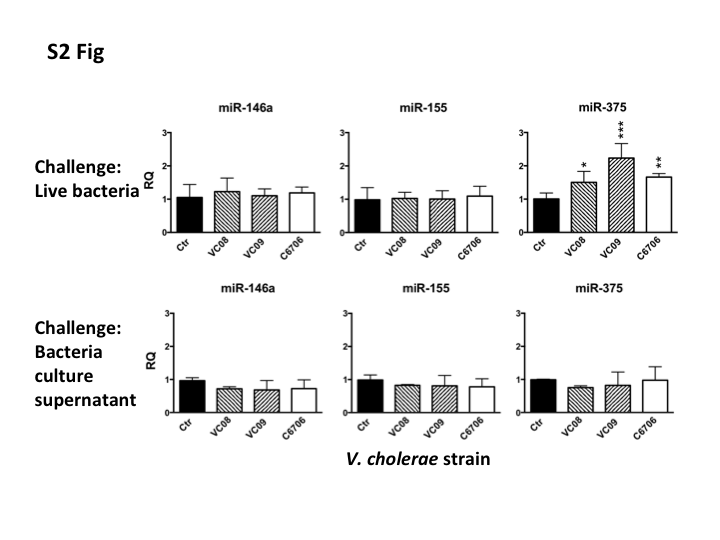

Supplement: S2 Fig — Tight monolayers of polarized T84 cells were challenged at the apical side with 105 bacteria of the V. cholerae O1 strain C6706 (C6706) and two V. cholerae O1 clinical isolates, one from patient VC08 (VC08) and one from patient VC09 (VC09), that had been grown in AKI broth overnight (upper row) or with culture supernatants from overnight cultures of the same bacterial strains in AKI (lower row). Levels of miR-146a, miR-155 and miR-375 were determined by real-time qRT-PCR and expressed as relative quantity (RQ) compared to the median Δct of tight monolayers incubated in parallel to challenged monolayers with tissue culture medium without added bacteria (Ctr, upper row) or fresh AKI (Ctr, lower row). Bars indicate mean RQ + 1 SD of 4 tight monolayers for each challenge in the upper row and 3 in the lower row. Statistically significant differences from the control monolayers as determined by one-way ANOVA with Dunett's compensation for multiple comparisons, are shown. * P-value <0.05, ** P-value <0.01, *** P-value <0.001. (TIFF) [file pone.0173817.s002.tiff]
