# Supplementary material for: Induction of immunomodulatory miR-146a and miR-155 in small intestinal epithelium of Vibrio cholerae infected patients at acute stage of cholera
Source: PLoS One. 2017 Mar 20;12(3):e0173817. doi: 10.1371/journal.pone.0173817 (PMC5358779; doi:10.1371/journal.pone.0173817)
Supplement: S1 Table — (DOCX) [file pone.0173817.s003.docx]

**S1 Table. Expression levels of mRNAs for miR-146a target genes, chemokines and inflammatory cytokines in duodenal biopsies at acute compared to convalescent stage of disease in patients with *Vibrio cholerae* O1 infection.**

| mRNA species | Expression level at acute stage  (Δct)^§^ |
| --- | --- |
|  | *miR-146a targets* |
| IRAK1 | 4.4 (3.7–4.6)° |
| TRAF6 | 4.3 (4.1–4.9) |
| CARD10 | 9.6 (8.6–9.9) |
|  | *Inflammasome cytokines* |
| IL-1β | 13.8 (12.0–15.0) |
| IL-18 | 15.2 (13.9–16.1) |
|  | *Chemokines* |
| CXCL9 | 10.8 (9.1–13.0) |
| CXCL10 | 13.4 (11.4–15.8) |
| CXCL11 | 17.2 (14.6–18.5) |
|  | (mRNA copies/18S rRNA U) ^§§^ |
| IL-8 | 1.45 (0.75–4.05) |
| CX3CL1 | 0.089 (0.056–0.17) |
|  | *Pro-inflammatory cytokines expressed in  epithelial cells and macrophages* |
| TNF-α | 0.93 (0.55-1.77) |
| IL-6 | 0.055 (0.016–0.20) |
|  | *Pro-inflammatory cytokines expressed in lymphocytes* |
| IL-17A | 0.34 (0.27–0.47) |
| IFN-γ | 0.086 (0.056–0.29) |

^§^ Δct: Ct-value for the mRNA species of interest minus ct-value of 18S rRNA yielding a scale from -5 to 22 where -5 is very high expression level and 22 is undetectable amounts.

° Median and interquartile range (IQR) from the 25^th^ to the 75^th^ percentile.

^§§^ Amounts of mRNA copies and 18S rRNA units were determined by real-time qRT-PCR assays with serially diluted external RNA copy standards.
