# Supplementary material for: Induction of immunomodulatory miR-146a and miR-155 in small intestinal epithelium of Vibrio cholerae infected patients at acute stage of cholera
Source: PLoS One. 2017 Mar 20;12(3):e0173817. doi: 10.1371/journal.pone.0173817 (PMC5358779; doi:10.1371/journal.pone.0173817)
Supplement: S1 File — (DOCX) [file pone.0173817.s004.docx]

**Supplementary Material**

**Supplementary Materials and Methods**

**Western blot analysis**

C6706 wild-type, C6706 cholera toxin (CT) deletion mutant [1], C6706 *Vibrio cholerae* cytolysin (VCC) deletion mutant (BML30)[2] and the *V. cholerae* O1 clinical isolates from patient VC08 (VC08) and patient VC09 (VC09) were grown over-night in AKI broth to OD_600nm_ 2.0 in a water bath shaker at 37°C. Bacteria cells were harvested by centrifugation at 14,000 rpm for 3 min at 4°C. The supernatant sample was precipitated 1:4 with 50% (w/v) trichloroacetic acid and incubated on ice for 15 minutes and subsequently centrifuged at 14,000 rpm for 15 minutes at 4°C. The pellet was washed twice with ice-cold PBS and resuspended in 20 mM Tris-HCl (pH 8.0) buffer. Protein samples were denatured in sample buffer containing 10% glycerol, 0.05% bromophenol blue, 2% SDS, 5% 2-mercaptoethanol, and 10 mM Tris-HCl, pH 6.8, and resolved by 13.5% SDS-PAGE with a discontinuous buffer system at a constant voltage of 60 V for the stacking gel and 120 V for the resolving gel. The proteins in the gel were transferred to a polyvinylidene difluoride membrane (Millipore) in standard transfer buffer with a Bio-Rad semidry transfer system at 23 V for 35 min. After the transfer was completed, the membrane was blocked with 5% skim milk in phosphate-buffered saline (PBS; pH 7.2) containing 0.05% Tween-20 at 4°C overnight. The immunoblot membrane was incubated with 1:10,000 dilutions of anti-*Vibrio cholerae* cytolysin (VCC) polyclonal antiserum [1, 2] or 1:5,000 dilutions of anti-cholera toxin (CT) polyclonal antiserum (Sigma-Aldrich; Product number-C3062) as a primary antibody for 1 hr. Horse radish peroxidase conjugated goat anti-rabbit IgG antibody (AgriSera) was used as a secondary antibody at a final dilution of 1:25,000. The ECL^+^chemiluminescence system (GE Healthcare) was used to detect immuno-reaction bands that were recorded using a Fluor-S MultiImager (BioRad) and by autoradiography.

**Supplementary References**

1. Skorupski K and Taylor RK. Cyclic AMP and its receptor protein negatively regulate the coordinate expression of cholera toxin and toxin-coregulated pilus in *Vibrio cholera*. Proc Natl Acad Sci USA 1997; 94(1): 265-270.

2. Ou G, Rompikuntal PK, Bitar A, Lindmark B, Vaitkevicius K, Bhakdi S, et al. *Vibrio cholerae* cytolysin causes an inflammatory response in human intestinal epithelial cells that is modulated by the PrtV protease. PLoS ONE 2009; 4(11): e7806.
